# Reduction of the acquisition time needed to obtain somatosensory evoked potentials by estimation of the required averaging sweep count by an algorithm

**DOI:** 10.1007/s10877-024-01217-3

**Published:** 2024-09-11

**Authors:** Clemens Bothe, Charlotte Winterling, Kai Berndt, Hajrullah Ahmeti, Alina Balandin, Markus Steinfath, Ann-Kristin Helmers, Axel Fudickar

**Affiliations:** University Hospital Schleswig-Holstein, Campus Kiel, Kiel, Germany

**Keywords:** Somatosensory evoked potentials, General anesthesia, Equipment and supplies, Electroencephalography, Monitoring

## Abstract

Somatosensory evoked potentials are frequently acquired by stimulation of the median or tibial nerves (mSEPs and tSEPs) for intraoperative monitoring of sensory pathways. Due to their low amplitudes it is common practice to average 200 or more sweeps to discern the evoked potentials from the background EEG. The aim of this study was to investigate if an algorithm designed to determine the lowest sweep count needed to obtain reproducible evoked potentials in each patient significantly reduces the median necessary sweep count to under 200. 30 patients undergoing spinal surgery at the Department of Neurosurgery were included in the study. Beginning with a sweep count of 200 an algorithm was designed to determine the lowest sweep count that yielded reproducible evoked potentials in each patient. By this algorithm the minimal sweep count was determined in 15 patients for mSEPs and in 15 patients for tSEPs. The required sweep count was below 200 in 14 of 15 patients for mSEPs (93.3%) with a mean sweep count of 56 ± 51. For tSEPs the sweep count was below 200 in 11 of 15 patients (73.3%) with a mean sweep count of 106 ± 70 (mean ± SD). The calculated mean time to average the potentials could thereby be reduced from 48.8s to 13.7s for mSEPs and from 48.8s to 25.9s for tSEPs. The proposed algorithm allowed sweep count and acquisition time reduction in roughly 90% of all patients for mSEPs and in 70% of all patients for tSEPs.

## Introduction

Monitoring of the nervous system is essential during many neurosurgical procedures at vulnerable sites or during vascular surgery with risk of cerebral or spinal hypoperfusion. These procedures include surgery of the spinal column and intracranial structures as well as carotid endarterectomy or replacement of the descending thoracic aorta. Somatosensory evoked potentials (SEPs) after electrostimulation of the median nerve (mSEPs) or the tibial nerve (tSEPs) can be used to monitor the functional integrity of the corresponding sensory neuronal paths during such procedures. Decrease of amplitudes or prolongation of SEPs-latencies are signs of functional impairment of neural structures in peripheral nerves, the spinal cord, the brainstem or the sensory cortex. This can be due to direct mechanical irritation, hypoperfusion, hypoxia or other pathological factors. SEPs enable monitoring of more brain areas than near infrared spectroscopy (NIRS) and have a better sensitivity for impairment of subcortical structures than EEG [1].

Due to the low amplitude of SEPs in comparison to the background activity of spontaneous EEG usually 200 or more single sweeps of SEPs are averaged to discern SEPs from background noise. This averaging process is time consuming and responsible for a considerable time delay when obtaining SEPs [2]. Hence, acceleration of averaging would abbreviate the process to obtain reliable SEPs measurements. Clinical observations suggest that averaging sweep counts lower than 200 may be sufficient for reliable and reproducible acquisition of SEPs in many patients [3].

The aim of this study was to investigate if it is possible to systematically reduce the sweep counts required for averaging of mSEPs and tSEPs by using an algorithm designed to determine the minimally required sweep count for each patient. Consequently, this algorithm could be clinically applied as it is or integrated into commercial neuromonitoring devices to find the required sweep count for every patient.

## Materials and methods

The study was approved by the Ethics Committee of the University Hospital Schleswig-Holstein, Campus Kiel (Chair: Prof. Dr. med. H.M. Mehdorn, Arnold-Heller-Str. 3/Haus U27, 24105 Kiel; Number of Processing: AZ D 573/17). The study was registered in the German Clinical Trials Register (DRKS00013669).

After preoxygenation with 100% oxygen, anesthesia was induced with propofol injection (1.5–2.5 mg/kg) and remifentanil infusion (0.2–0.6 µg/kg/min). For maintenance of anesthesia the remifentanil infusion was continued and propofol was infused at 4–12 mg/kg/h. Muscle relaxation was achieved with intravenous rocuronium (0.3–0.6 mg/kg). mSEPs and tSEPs were derived after steady state anesthesia was achieved. All measurements were performed using a NIM-ECLIPSE^®^ E4 Monitoring-System (Medtronic, Dublin, Ireland).

After preparing the skin with peeling paste (Everi Paste, Spes medica, Genua, Italy) and applying a conductive paste (Elefix paste for EEG, Nihon Kohden, Tokyo, Japan) EEG electrodes were placed according to the international 10–20 system of electrode placement. Electrodes were located at Fz, C3` and C4` for mSEPs and at Fz and Cz` for tSEPs. Filter setting was 30–500 Hz and stimulation frequency was 4.1 Hz. The peaks used to assess reproducibility were N20 and N37 and the amplitudes used to assess reproducibility were measured between N20 and P25 and between N37 and P40 for mSEPs and tSEPs, respectively. Measurements were facilitated by a cursor function with automatic amplitude and latency calculation provided by the NIM-ECLIPSE. A ground electrode was fixed on the chest after preparation of the skin as described above. Electrode impedances were below 5 kΩ for all leads.

Stimulation of the median and tibial nerve was achieved by using surface stimulation electrodes above the median nerve at the level of the carpal tunnel for mSEPs on both arms and the tibial nerve at the ankle for tSEPs on both legs.

Stimulation was performed after induction of anesthesia including the application of the muscle relaxant rocuronium (0.3–0.6 mg/kg). Current strength was 15 mA for mSEPs and 30 mA for tSEPs; impulse duration was 200µs. NIM-ECLIPSE measures and displays automatically, if the applied stimulation intensities are equal to the preset intensities. Thus, sufficient stimulation current was ensured in all patients.

These stimulator settings resulted in motor response (Adduction of the thumb for MSEPs and flexion of the forefoot for TSEPs) and SEPs in all study patients despite muscle relaxation during induction of anesthesia. Thus, further increase of stimulation impulse intensity was not necessary in any study patient, but was envisaged in principle if motor response or SEPs would have been absent. Thus, sufficient stimulation effect was verified by the presence of motor response and SEPs during baseline measurements.

**Fig. 1:**
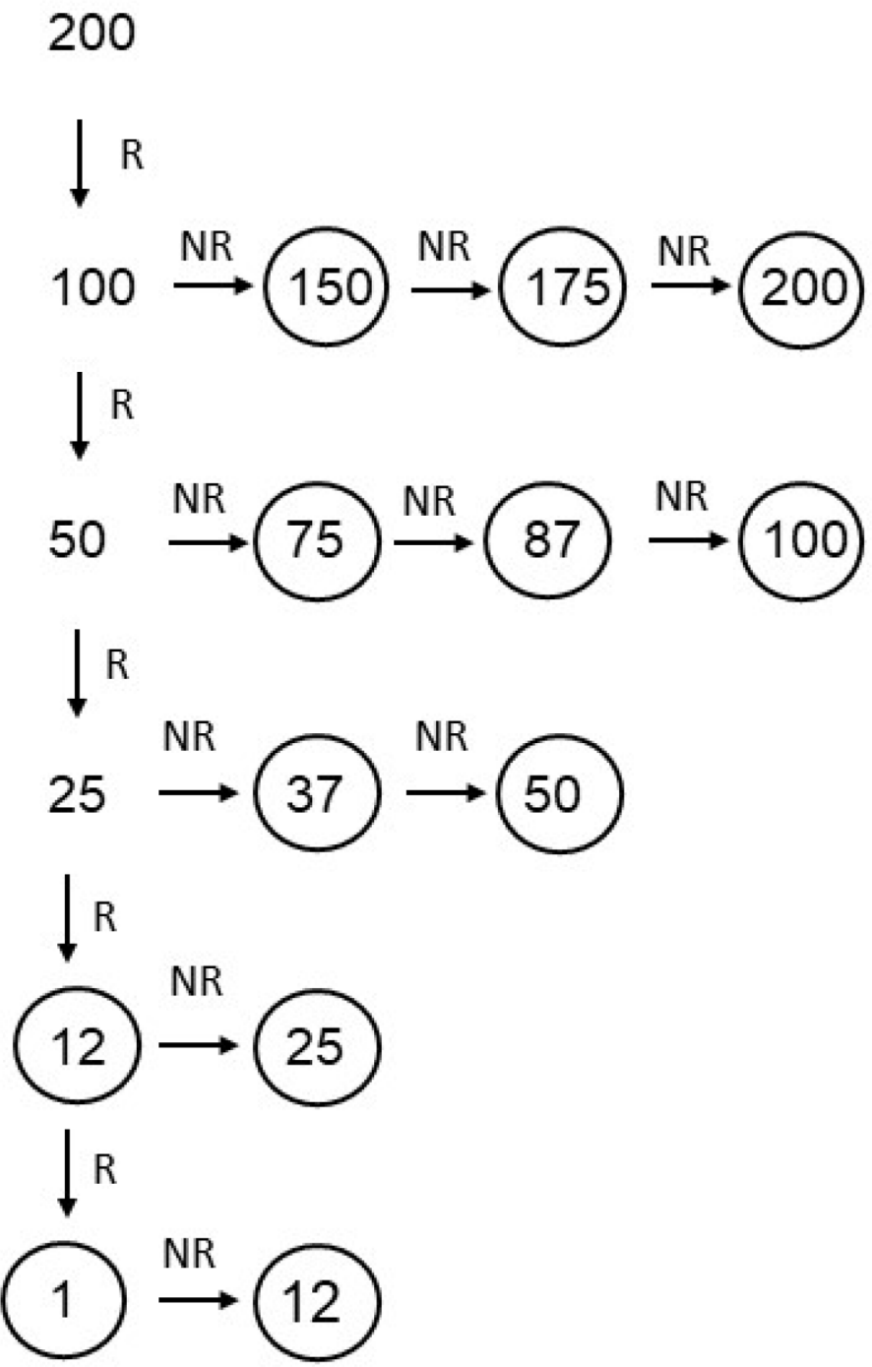
Sweep count reduction algorithm. *Legend* First, five measurements with sweep count 200 were performed to confirm the feasibility of reproducible SEPs with standard sweep count. Reproducibility was defined as a difference of less than 20% between amplitudes and less than 0.5 ms between latencies between measurements. If reproducible SEPs could be obtained with sweep count 200, the sweep count was reduced to 100. If SEPs were not reproducible with sweep count 100, the sweep count was increased in the steps defined in Fig. 1 until the SEPs were reproducible again. If SEPs were reproducible with sweep count 100, the sweep count was further reduced to 50. This procedure of sweep count reduction and increase, if the reduction resulted in no reproducible SEPs was performed following the algorithm displayed in Fig. 1 in the same way until the lowest reproducible circled sweep count was reached. The lowest reproducible circled sweep count was reached when no further reduction was possible without loss of reproducibility (R = reproducible, NR = not reproducible)

An algorithm was designed to determine the minimally required sweep count for each patient and applied in each patient (Fig. 1): First, five baseline measurements with sweep count 200 were performed to confirm the feasibility of reproducible SEPs with standard sweep count. Reproducibility was defined as a difference of less than 20% between amplitudes and less than 0.5ms between latencies between two measurements. If reproducible SEPs could be obtained with sweep count 200, the sweep count was reduced to 100. If SEPs were not reproducible with sweep count 100, the sweep count was increased in the steps defined in Fig. 1 until the SEPs were reproducible again. If SEPs were reproducible with sweep count 100, the sweep count was further reduced to 50. This procedure of sweep count reduction and increase if the reduction resulted in irreproducible SEPs, was performed following the algorithm displayed in Fig. 1 in the same way until the lowest reproducible circled sweep count was reached. The lowest reproducible circled sweep count was reached when no further reduction was possible without loss of reproducibility. Thus, the lowest sweep count yielding reproducible potentials was found for each individual patient. Thereafter three more measurements were performed to calculate the precision of the measurements with the obtained minimal sweep count and to investigate if these precisions were significantly different from the precisions of the measurements with 200 sweep counts. The algorithm was applied manually and the NIM-ECLIPSE used to acquire SEPs data was not programmed to acquire data using the algorithm. However, a computer programmed version of the algorithm is feasible in principal.

### Statistical analysis

Due to missing preexisting studies the group size needed to obtain significant results with sufficient power could not be calculated by power analysis. The patient number was estimated according to a study by MacDonald et al., who showed in a study with 35 patients that it is possible to obtain reproducible somatosensory potentials with reduced sweep counts [4]. The patient numbers were limited to 15 patients for mSEPs and 15 patients for tSEPs, since highly significant results were obtained with these patient numbers.

Statistical analysis was performed with a commercially available software (GraphPad Prism, Version 5, GraphPad Software, San Francisco, CA, USA).

All results were tested for normality with the Shapiro-Wilk normality test. The significance of the difference between 200 and the mean minimally required sweep counts when using the algorithm was calculated with one sample t-test for mSEPs and tSEPs separately. Precision of SEPs for a sample of SEPs measurements was calculated as the standard deviation of the amplitudes of the measured SEPs in the sample divided by the square root of the number of measurements per sample. The precisions of the five measurements with 200 sweeps and three measurements with the obtained minimally required sweeps were calculated for each patient and the mean precisions of measurements with 200 sweeps compared to the precisions of measurements with the minimally required sweeps with Wilcoxon-Tests.

Two patients were excluded after the study after primary inclusion because SEPs measurement was impossible due to technical equipment problems.

## Results

10 males and 5 females were investigated with tSEPs and 7 males and 8 females were investigated with mSEPs. Mean age of the patients was 63.4 ± 16.3 years, mean height was 174.3 ± 12.0 cm and mean weight was 85.1 ± 22.0 kg (mean ± standard deviation). Body temperature was normal (36.5–37.5 °C). The patient’s health status was scored according to the American Society of Anesthesiologists (ASA) as ASA I in 3 patients, ASA II in 16 patients and ASA III in 11 patients (Table 1a and b).

**Table 1:** Demographic data and surgical procedures for mSEPs (a) and tSEPs (b) patients

**a)**	
Age (Years) Weight (Kg) Height (cm) Gender (male/female) ASA I/II/III Nucleotomy Foraminotomy Stabilization of the spinal column	60 ± 15.7 79 ± 19.7 172 ± 13.1 7/9 3/9/3 13 1 1
**b**)	
Age (Years) Weight (Kg) Height (cm) Gender (male/female) ASA I/II/III Stabilization after fracture of vertebrae Nucleotomy Foraminotomy	66,8 ± 14.6 91,8 ± 22.9 176,3 ± 11.13 10/5 0/7/8 6 5 3

*Legend* (a) Demographic data and surgical procedures for measurements of mSEPs patients. (b) Demographic data and surgical procedures for measurements of tSEPs patients

Surgical procedures were nucleotomies (*N* = 18), stabilization after fractures of vertebrae (*N* = 7), foraminotomies (*N* = 4) and dorsal stabilization of the vertebral column due to rheumatic bone demineralization (*N* = 1).

### mSEPs

**Fig. 2:**
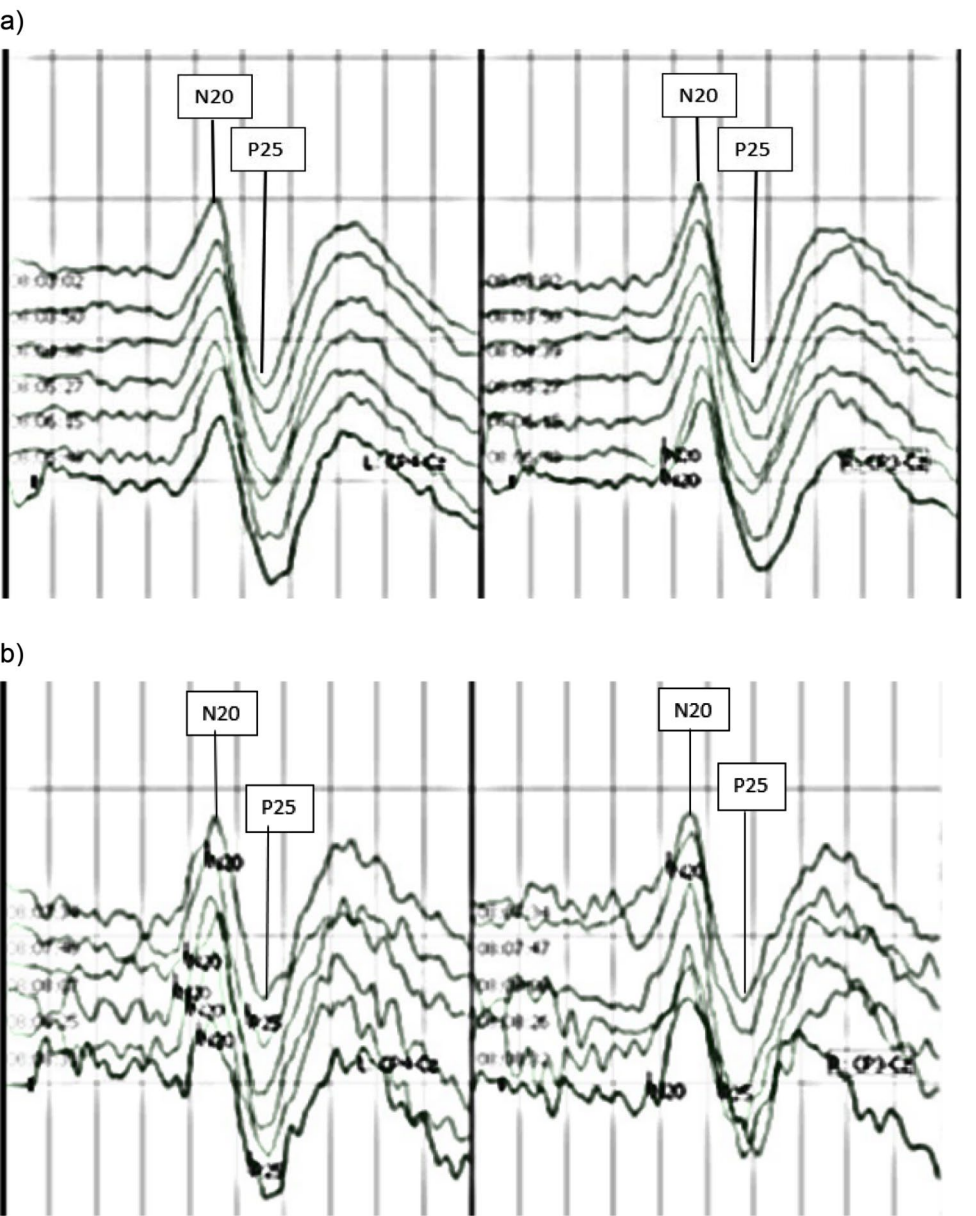
mSEPs obtained from one single patient with particularly low minimal sweep count after averaging 200 sweeps (**a**) and 25 sweeps (**b**) while applying the algorithm described in Fig. 1. *Legend* (**a**) mSEPs obtained by averaging 200 sweeps. The mSEPs are clearly identifiable. (**b**) mSEPs obtained by averaging 25 sweeps from the same patient as in a). The mSEPs are still identifiable

Sweep counts could be reduced in 14 of 15 patients for mSEPs (93.3%). Mean lowest sweep count yielding reproducible results was 56 ± 51 (mean ± standard deviation). The reduction of sweep counts under 200 was significant for both sides (right side *p* = 0.0024; left side *p* = 0.0024). Precision for measurements with 200 sweeps was 0.0047 ± 0.003 on the right side and 0.06 ± 0.029 on the left side. For the lowest sweep count yielding reproducible results the precision was 0.034 ± 0.033 on the right side and 0.055 ± 0.067 on the left side. Differences between measurement precisions with lowest sweep counts yielding reproducible results and measurement precisions with 200 sweeps were not significant on both sides (right side *p* = 0.068; left side *p* = 0.11). Figure 2a and b show mSEPs after averaging 200 and 25 sweeps obtained from one single patient with particularly low minimal sweep count.

### tSEPs

**Fig. 3:**
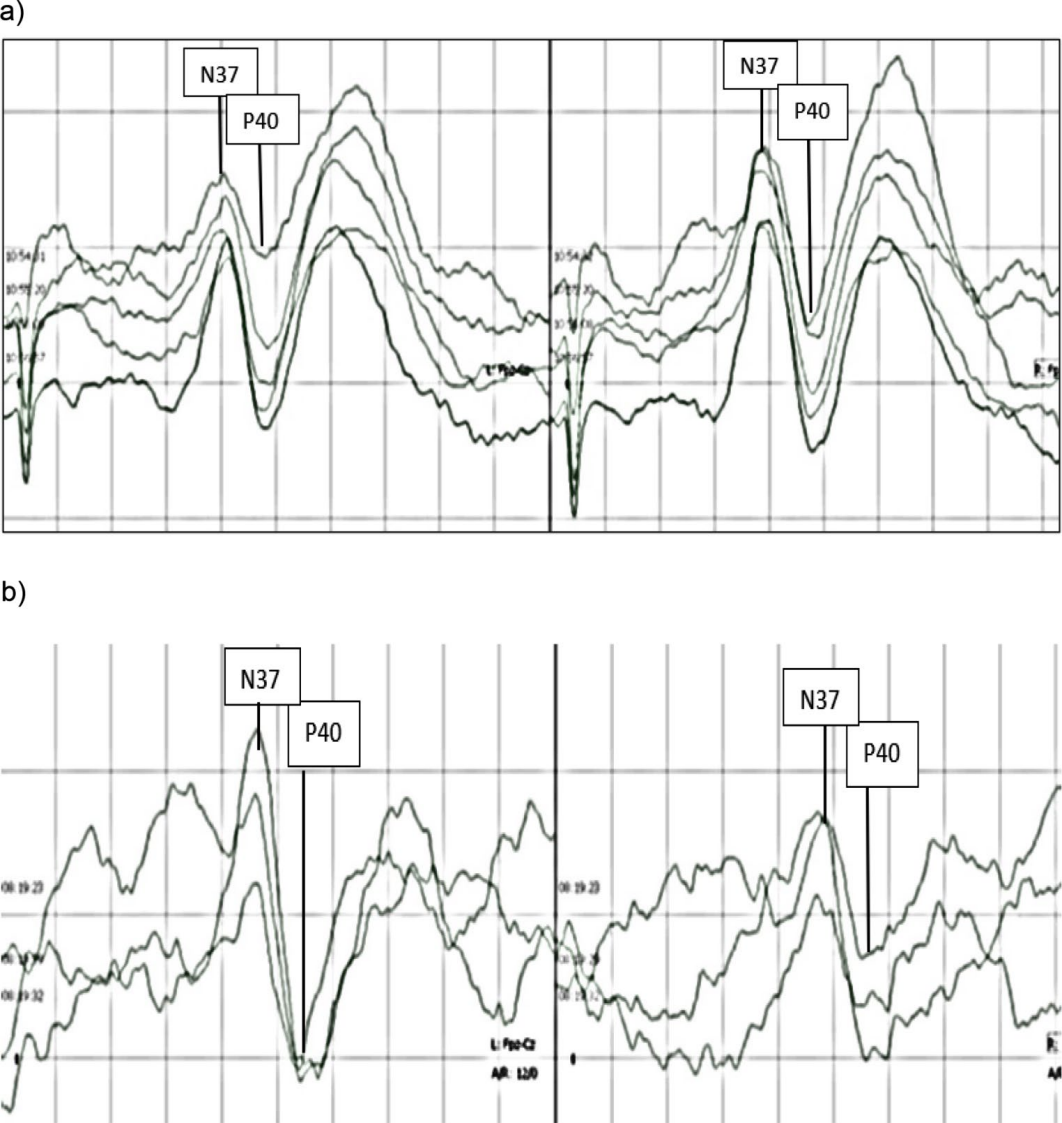
tSEPs obtained from one single patient with particularly low minimal sweep count after averaging 200 sweeps (**a**) and 12 sweeps (**b**) while applying the algorithm described in Fig. 1. *Legend* (**a**) tSEPs obtained by averaging 200 sweeps. The positive amplitude N37 and the following negative amplitude P40 are clearly distinguishable frombaseline. (**b**) tSEPs obtained by averaging 12 sweeps from the same patient as in a). The amplitudes are still distinguishable from baseline, but the noise-level is increased

tSEPs sweep counts could be reduced in 11 of 15 patients (73.3%). The mean lowest sweep count yielding reproducible results was 106 ± 70 (mean ± standard deviation). The reduction of sweep counts under 200 was significant for both extremities (right sided *p* = 0.0056; left sided *p* = 0.0037). Calculated precision for measurement with 200 sweeps was 0.064 ± 0.044 on the right side and 0.068 ± 0,057 on the left side. Precision for lowest possible sweep counts was 0.063 ± 0,047 on the right side and 0,057 ± 0,046 on the left side. Difference in precision for measurement with 200 sweep counts and lowest sweep counts yielding reproducible results was not significant on both sides (right side *p* = 1; left side *p* = 0,2412). Figure 3a and b show mSEPs after averaging 200 and 25 sweeps obtained from one single patient with particularly low minimal sweep count.

### Comparison of mSEPs with tSEPs

**Fig. 4:**
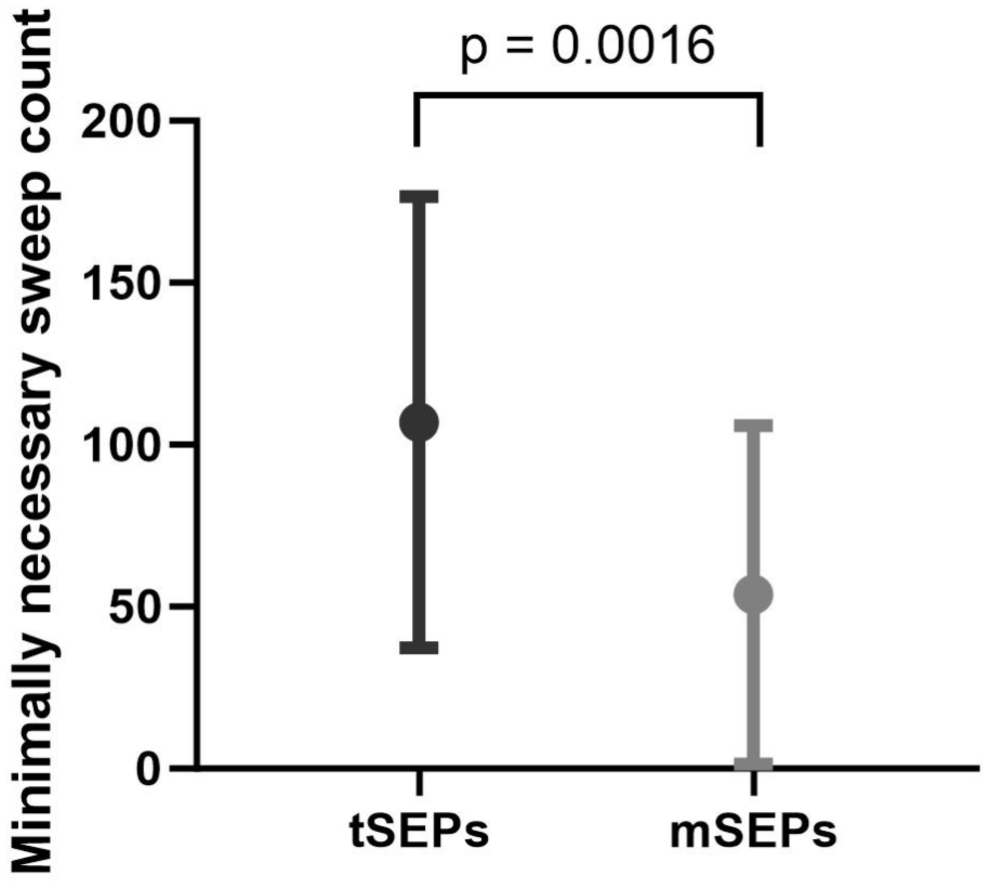
Comparison of minimally necessary sweep counts for mSEPs and tSEPs. *Legend* Minimal sweep count numbers required to obtain somatosensory evoked potentials after stimulation of the tibial nerve (tSEPs, 106 ± 70 (mean ± standard deviation), *N* = 15, black symbol for mean (●) and black error bars for standard deviations (^┬^, _┴_) and after stimulation of the median nerve (mSEPs, 56 ± 51 (mean ± standard deviation), *N* = 15, grey symbol for mean (●) and grey error bars for standard deviations (^┬^, _┴_). The difference between the means is significant (*p* < 0.0016)

Reduction of sweep counts was feasible in more patients for mSEPs than for tSEPs and significantly less sweep counts were necessary for mSEPs than for tSEPs (56 ± 51 for mSEPs vs. 106 ± 70 for tSEPs; *p* = 0.0016 (mean ± standard deviation)). The difference in the number of required sweeps while using the proposed algorithm is shown in Fig. 4.

### Acquisition time

The difference in the number of required sweeps resulted in longer acquisition times after reduction of the sweep counts for tSEPs in comparison to mSEPs. The times needed to obtain reproducible potentials were calculated from sweep counts and stimulation frequencies as follows: Measurement time = sweep counts (1/stimulation frequency). Hence, for measurement of tSEPs and mSEPs the calculated acquisition time for averaging 200 sweeps was 48.8s. Accordingly the measured time needed for acquisition of mSEPs and tSEPs with averaging 200 sweeps was 48s. The acquisition time was measured by measuring the time from starting a sweep until occurrence of the averaged curve on the monitor.

**Fig. 5:**
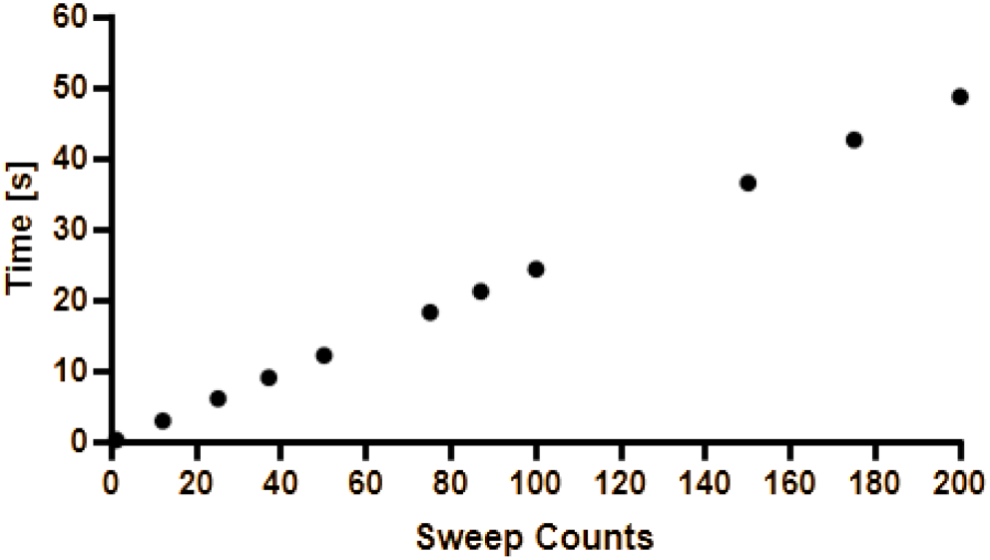
Calculated time needed for each sweep count step. Calculated time needed for each sweep count (y-axis) step in seconds (x-axis)

For the mean lowest sweep count of mSEPs after reduction by the algorithm (56 sweeps) 13.7s were calculated and for the mean lowest sweep count of tSEPs after reduction by the algorithm (106 sweeps) 25.9s were calculated. The calculated acquisition times are shown in Fig. 5 for each sweep count. Thus, this technique could detect a change in SEPs amplitude earlier than the standard method, because the time to average less than 200 single sweeps is shorter than the time to average 200 single sweeps or more as recommended as standard averaging technique if the same stimulation frequency is applied.

## Discussion

The most important findings of our study were:

1. Reduction of sweep counts required for measurement of reproducible mSEPs and tSEPs was feasible for 93.3% of all mSEPs measurements and 73.3% of all tSEPs measurements.
2. The calculated time needed for the acquisition of SEPs could be reduced from 48.8s to 13.7s for mSEPs measurements and from 48.8s to 25.9s for tSEPs measurements by reducing the sweep counts according to the above described algorithm.
3. Reduction of sweep counts was feasible in more patients for mSEPs measurements (14) than for tSEPs measurements (11) and significantly less sweep counts were necessary for mSEPs measurements than for tSEPs measurements (56 ± 51 for mSEPs; 106 ± 70 for tSEPs; *p* = 0.0016 (mean ± standard deviation)).

The first documented measurement of SEPs after stimulation of peripheral nerves was reported by Dawson in 1947 [5]. The technique was later adopted into clinical practice when it was utilized for non-invasive intraoperative neuromonitoring and SEPs largely replaced the “wake-up Test” for assessing motor function during surgery. For the “Wake-up Test”, the patients were allowed to wake up from anesthesia during surgery and asked to perform movement tests. This procedure was hazardous and was usually performed only once during surgery [6].

By contrast, the introduction of SEPs in intraoperative neuromonitoring rendered nearly continuous and safe monitoring possible. Sensitivity of SEPs for functional impairment ranges from 80 to 100% and specificity ranges from 98.8 to 99.2% [7–9]. During surgery of the descendent aorta a permanent loss of tSEPs resulting from spinal ischemia is a good predictor for postoperative paraplegia. If tSEPs recur after reperfusion of the spinal cord within 40 min, the neurological outcome improves [9, 10].

Optimal signal-to-noise ratio is essential for reproducibility and determines the sweep count needed for acquisition of SEPs with sufficient sensitivity to impairment of neural function. MacDonald et al. showed that the location of SEPs derivations has a significant influence on signal quality. He determined the sweep counts necessary to obtain the potentials P31 and P37 of tSEPs with acceptable signal-to-noise ratio dependent on different electrode locations. The ratio between amplitudes of potentials and background noise was compared and used as a degree of derivation quality. He showed that 64 sweeps were sufficient to obtain the P37 potential of tSEPs from the location with the best signal-to-noise ratio (CPc-CPz), while in general 128 sweeps were necessary [11]. For mSEPs only 50 sweeps were sufficient to obtain reproducible potentials [4].

Hence, the use of multiple electrodes aiming at the detection of the location with the lowest sweep count needed to obtain reproducible SEPs could result in improving the reproducibility of evoked potentials and reduction of data acquisition time in principle. However, this approach requires additional setup time for placing the electrodes and then time to assess the recordings, time which may not be available particularly if multiple monitoring modalities are being utilized. As a result, using our algorithm that has been proposed in conjunction with standard recording sites may achieve the same end without any additional burdens. These considerations are consistent with our results that yielded mean minimal sweep counts for acquisition of mSEPs and tSEPs (56 ± 51 for mSEPs, 106 ± 70 for tSEPs) similar to those obtained by McDonald et al.

Due to the risk of cerebrospinal lesions limited to the motor or the sensory tract, a combination of transcranial motor evoked potentials (tceMEP) and SEPs is recommended for intraoperative neuromonitoring (IONM) of the spinal cord and vascular surgeries [12, 1].

The internationally accepted criteria suggesting functional impairment of the posterior ascending tract in the spinal column is a reduction of the tSEPs amplitude by 50%. However, some authors propose an amplitude reduction of 30% as criterion due to false negative results [7, 13]. Even after optimizing surgical techniques and IONM modalities 0.04–0.06% of postoperative neurological deficits are not predicted intraoperatively [14–16].

IONM with SEPs is limited by some concomitant diseases. Polyneuropathies like diabetic polyneuropathy can decrease nerve conduction velocity [1, 17].

Hence, signal quality varies between patients and systematical and individual reduction of sweep counts by using an algorithm as described in our study may be reasonable and may accelerate detection and reporting of imminent neuronal damage to the surgeon in a subgroup. The reaction time to reversible functional impairments may become reduced in this subgroup and thus the incidence of postoperative neurological deficits may decrease.

Due to their different stimulation sites and neuronal pathways, signal qualities of mSEPs and tSEPs are impaired by anatomical, physiological and technical confounders to a different degree. mSEPs are elicited by stimulation of the median nerve at the wrist; tSEPs are elicited by stimulation of the tibial nerve at the ankle. Thus, the signal pathway of tSEPs from the stimulation site to the sensory cortex is roughly twice as long as the signal pathway of mSEPs. Hence, the probability of interference with noise is higher for tSEPs. Moreover, due to longer sensory nerve fibers and slightly different conduction velocities and stimulation times of single axons the sum potentials of tSEPs flatten and broaden during neuronal signal conduction, which impairs readability. Concordant with the highly differentiated sensory function of the hand, its representation on the primary sensory cortex is relatively high in comparison to other extremities or the torso [18]. As a consequence, the amplitudes of mSEPs tend to be higher in comparison to the amplitudes of tSEPs.

Consistent with these theoretical considerations we found that significantly fewer sweep counts are needed to measure reproducible mSEPs potentials in comparison to tSEPs.

### Limitations

The standard averaging process is time consuming and responsible for a considerable time delay when obtaining SEPs, thus motivating the design of the investigated algorithm. However, the importance of this time delay in terms of patient outcome remains questionable, because it has not been proven that this time delay contributes meaningfully to patient outcomes. Standard deviations of the results are large and preclude a general recommendation of lower sweep counts practicable for all patients. Changing the criteria for how reproducibility is defined to a difference of less than 10% as criterion for reproducibility might change the results when using the algorithm to a minor reduction of acquisition time, because loss of reproducibility would occur at less prominent differences and thus the algorithm would end at higher sweep counts and therewith longer acquisition times. However, the algorithm allows to reduce sweep counts in most patients thus rendering it practicable as a tool to determine the minimally required sweep count specific for each single patient. Changes in blood pressure, temperature, anesthetic depth, and other factors have to be considered when evaluating evoked potentials in general. As a caution for readers, keeping these parameters as steady as possible is a basic prerequisite of any potential clinical application of the algorithm and the algorithms reliability during surgery has yet to be verified during intraoperative monitoring.

Stimulation intensities were set at 15 mA for MSEPs and 30 mA for TSEPs. During our clinical practice of acquiring SEPs for intraoperative neuromonitoring using the NIM-Eclipse monitoring system the described stimulator settings result regularly in motor responses and SEPs. Hence, we used the same settings for our study and obtained motor responses and SEPs in all patients. Determining supramaximal stimulus intensity for each patient by beginning at very low stimulation intensities followed by stepwise increases may have resulted in different stimulation intensities in each study patient, but would have been more time consuming.

## Conclusion

It is possible to use the above described algorithm to significantly reduce the sweep counts necessary to obtain reproducible measurements of mSEPs and tSEPs. Sweep counts could be reduced from 200 to 56 ± 51 for mSEPs and to 106 ± 70 for tSEPs (mean ± standard deviation). The algorithm´s reliability during surgery has yet to be verified during intraoperative monitoring.

## Data Availability

No datasets were generated or analysed during the current study.

## References

[1] DGAI, SGAR, DGTHG, Neuromonitoring in der Kardioanästhesie: Gemeinsame Stellungnahme der Deutschen Gesellschaft für Anästhesiologie und Intensivmedizin (DGAI), Schweizerischen Gesellschaft für Anästhesiologie und Reanimation (SGAR) und Deutschen Gesellschaft für Thorax-, Herz- und Gefäßchirurgie (DGTHG). Z Herz- Thorax- Gefäßchir, Bd. 28, Nr. 6, S. 430–447, Dez. 2014, 10.1007/s00398-014-1125-4.

[2] MacDonald DE. Recommendations of the international society of intraoperative neurophysiology for intraoperative somatosensory evoked potentials. Clin Neurophysiol. S. 19;2019. 10.1016/j.clinph.2018.10.008 30470625

[3] Fudickar A, Maurer E, Linstedt U, Dinkel M, Scholz J, Tonner PH. Elektroenzephalogramm und evozierte Potenziale in der Intensivmedizin. Anästh Intensivmed. S. 8;2007.

[4] MacDonald DB, Al-Zayed Z, Stigsby B, Al-Homoud I. Median somatosensory evoked potential intraoperative monitoring: Recommendations based on signal-to-noise ratio analysis. Clin Neurophysiol. Bd. 120, Nr. 2, S. 315–328, Feb. 2009. 10.1016/j.clinph.2008.10.154. 10.1016/j.clinph.2008.10.154 19111507

[5] Dawson GD. Cerebral responses to electrical stimulation of peripheral nerve in man. J Neurol, Neurosurg Psych. Bd. 10, Nr. 3, S. 134–140, Aug. 1947. 10.1136/jnnp.10.3.134. 10.1136/jnnp.10.3.134 PMC497105 21610884

[6] Galloway GM, Nuwer MR, Lopez JR, Zamel KM. Intraoperative Neurophysiol Monitoring. 1. Aufl. Cambridge University Press, 2010. 10.1017/CBO9780511777950.

[7] Shilian P, Zada G, Kim AC Gonzalez AA. Overview of intraoperative neurophysiological monitoring during spine surgery. J Clin Neurophysiol. Bd. 33, Nr. 4, S. 333–339, Aug. 2016. 10.1097/WNP.0000000000000132. 10.1097/WNP.0000000000000132 25233250

[8] Wiedemayer H, Sandalcioglu IE, Armbruster W, Regel J, Schaefer H, Stolke D. False negative findings in intraoperative SEPs monitoring: analysis of 658 consecutive neurosurgical cases and review of published reports. J Neurol Neurosurg Psychiatry. Feb. 2004;75(2):280–6. 10.1136/jnnp.2002.008904. PMC1738927 14742605

[9] McGarvey ML. In: Fleisher LA, editor. und A. Joshi. 47 - does neurologic electrophysiologic monitoring affect outcome? In evidence-based practice of Anesthesiology (Fourth Edition). Philadelphia: Elsevier; 2023. pp. 417–29. 10.1016/B978-0-323-77846-6.00047-1.

[10] Sloan TB. Jameson. Electrophysiologic monitoring during surgery to Repair the Thoraco-Abdominal Aorta. J Clin Neurophysiol. Aug. 2007;24(4):316–27. 10.1097/WNP.0b013e31811ebc66. 10.1097/WNP.0b013e31811ebc66 17938600

[11] MacDonald DB, Al Zayed Z, Stigsby B. Aug. Tibial somatosensory evoked potential intraoperative monitoring: Recommendations based on signal to noise ratio analysis of popliteal fossa, optimized P37, standard P37, and P31 potentials. Clinical Neurophysiology, Bd. 116, Nr. 8, S. 1858–1869, 2005, 10.1016/j.clinph.2005.04.018 10.1016/j.clinph.2005.04.018 16005261

[12] Azabou E, Delage J-M, Hennig M, Macadoux G, Lofaso F. und C. Garreau de Loubresse. [Selective and non-invasive monitoring of the posterior columns and pyramidal tract during surgery of the spine and spinal cord]. Rev Neurol (Paris), Bd. 171, Nr. 8–9, S. 646–654, SEPs. 2015, 10.1016/j.neurol.2015.04.006 10.1016/j.neurol.2015.04.006 26321313

[13] Liu Q, Wang Q, Liu H, Wu WKK. und M. T. V. Chan. Warning criteria for intraoperative neurophysiologic monitoring. Curr Opin Anaesthesiol, Bd. 30, Nr. 5, S. 557–562, Okt. 2017, 10.1097/ACO.0000000000000505 10.1097/ACO.0000000000000505 28719456

[14] Nuwer MR, Dawson EG, Carlson LG, Kanim LE. Sherman. Somatosensory evoked potential spinal cord monitoring reduces neurologic deficits after scoliosis surgery: results of a large multicenter survey. Electroencephalogr Clin Neurophysiol. Jan. 1995;96(1):6–11. 10.1016/0013-4694(94)00235-d. 10.1016/0013-4694(94)00235-d 7530190

[15] Tamkus AA, Rice KS, McCaffrey MT. Perils of intraoperative neurophysiological monitoring: analysis of false-negative results in spine surgeries. Spine J. Feb. 2018;18:276–84. 10.1016/j.spinee.2017.07.005. 10.1016/j.spinee.2017.07.005 28713053

[16] Halsey MF, Myung KS, Ghag A, Vitale MG, Newton PO. Aug., und M. De Kleuver. Neurophysiological monitoring of spinal cord function during spinal deformity surgery: 2020 SRS neuromonitoring information statement. Spine Deform, Bd. 8, Nr. 4, S. 591–596, 2020, 10.1007/s43390-020-00140-2 10.1007/s43390-020-00140-2 32451978

[17] Ziegler D, Papanas N, Vinik AI, Shaw JE. Chapter 1 - Epidemiology of polyneuropathy in diabetes and prediabetes. in Handbook of Clinical Neurology, Bd. 126, D. W. Zochodne und R. A. Malik, Hrsg., in Diabetes and the Nervous System, vol. 126., Elsevier, 2014, S. 3–22. 10.1016/B978-0-444-53480-4.00001-1 10.1016/B978-0-444-53480-4.00001-1 25410210

[18] Lehmann-Horn F, Weber F. In: Nowak DA, editor. Funktionelle Neuroanatomie Der Hand. In Handfunktionsstörungen in Der Neurologie: Klinik Und Rehabilitation. Berlin, Heidelberg: Springer; 2011. pp. 13–23. 10.1007/978-3-642-17257-1_2.

